# Genes and miRNAs as Hurdles and Promoters of Corticospinal Tract Regeneration in Spinal Cord Injury

**DOI:** 10.3389/fcell.2021.748911

**Published:** 2021-10-15

**Authors:** Marina Boido, Alessandro Vercelli

**Affiliations:** Department of Neuroscience “Rita Levi Montalcini”, Neuroscience Institute Cavalieri Ottolenghi, University of Turin, Turin, Italy

**Keywords:** axonal regrowth, corticospinal tract (CST), RAGs, glial scar, developmental programs

## Abstract

Spinal cord injury (SCI) is a devastating lesion to the spinal cord, which determines the interruption of ascending/descending axonal tracts, the loss of supraspinal control of sensory-motor functions below the injured site, and severe autonomic dysfunctions, dramatically impacting the quality of life of the patients. After the acute inflammatory phase, the progressive formation of the astrocytic glial scar characterizes the acute-chronic phase: such scar represents one of the main obstacles to the axonal regeneration that, as known, is very limited in the central nervous system (CNS). Unfortunately, a cure for SCI is still lacking: the current clinical approaches are mainly based on early vertebral column stabilization, anti-inflammatory drug administration, and rehabilitation programs. However, new experimental therapeutic strategies are under investigation, one of which is to stimulate axonal regrowth and bypass the glial scar. One major issue in axonal regrowth consists of the different genetic programs, which characterize axonal development and maturation. Here, we will review the main hurdles that in adulthood limit axonal regeneration after SCI, describing the key genes, transcription factors, and miRNAs involved in these processes (seen their reciprocal influencing action), with particular attention to corticospinal motor neurons located in the sensory-motor cortex and subjected to axotomy in case of SCI. We will highlight the functional complexity of the neural regeneration programs. We will also discuss if specific axon growth programs, that undergo a physiological downregulation during CNS development, could be reactivated after a spinal cord trauma to sustain regrowth, representing a new potential therapeutic approach.

## Introduction

Worldwide, every year up to 500,000 people experience a spinal cord injury (SCI), which usually causes remarkable dysfunctions and disabilities, determining long-lasting and irreversible motor, sensory, and/or autonomic deficits (World Health Organization, 2013). This tragic condition also determines remarkable economic and social consequences (Thuret et al., 2006).

The pathophysiology of SCI is biphasic, consisting of a primary and a secondary phase, further divisible in other consecutive stages (i.e., immediate, acute, intermediate, and chronic stages). The primary phase involves the initial mechanical injury (compression, distraction, laceration, or transection of the spinal cord): it initiates a cascade of cellular and molecular escalating events, leading to the secondary injury phase. The first hours (immediate stage) are characterized by massive death of neurons and glia, axonal damage, spinal cord swelling, hemorrhage, and ischemia. During the following days/weeks (acute and intermediate stages), inflammation, excitotoxicity, demyelination, formation of the cystic cavity, and glial scar occur most frequently. The chronic phase, starting 6 months after SCI, is characterized by maturation/stabilization of the lesion, continued scar formation, development of cysts or syrinxes, and necrotic death (Vercelli and Boido, 2014). In the first weeks after injury, a spontaneous regenerative attempt can occur, even though it is not sufficient to support a functional recovery; however, in the last phase, the degenerative and inflammatory events become chronic and exacerbate the damage, making any effort to repair vain (Rowland et al., 2008).

Unfortunately, a cure for SCI is still lacking: the current clinical approaches are mainly based on early vertebral column stabilization, anti-inflammatory drug administration, and rehabilitation programs. However, new experimental therapeutic strategies are under investigation, to stimulate axonal regrowth and bypass the glial scar. Understanding the damage mechanisms and unraveling the intrinsic recovery potential of CNS (although limited) is pivotal to treat SCI. In this review, we will describe the key genes, transcription factors (TFs) and miRNAs, involved in axonal outgrowth and regeneration, whose activity can be intertwined and represent an intriguing therapeutic target for SCI.

## Learning From the Embryonic Development to Trigger Regeneration in Adulthood

**FIGURE 1:**
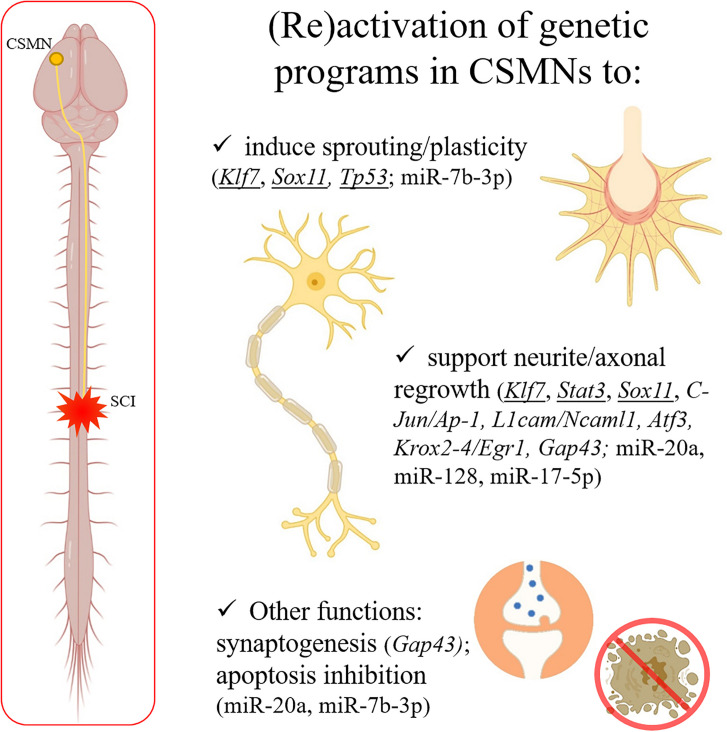
After a spinal trauma, CSMN axons can be severely damaged. CNS shows a limited capability in spontaneous regeneration after injury. Some well-known genes responsible for axonal outgrowth during the development (underlined in the figure) can be experimentally reactivated to support regeneration in the adulthood after SCI. Moreover, other genes and miRNAs (indicated in the panel) are emerging as interesting therapeutic targets, since able to induce sprouting and plasticity, support neurite/axonal regrowth, induce synaptogenesis, and/or inhibit apoptosis after a trauma in the adult CNS. Created with BioRender.com.

Motor disabilities following SCI are essentially due to axotomy affecting corticospinal motor neurons (CSMNs), whose cell body is located in the layer V of the motor and somatosensory cortical areas (often referred to as sensory-motor cortex) (Figure 1). The corticospinal tract (CST) is fundamental to regulate voluntary movements (Oudega and Perez, 2012).

The specification of CSMNs, also known as “upper motor neurons,” depends on genes that are progressively restricted and specific to this cell population. *CTIP2* is specifically expressed by CSMNs, and not from other pyramidal neurons (as callosal neurons), despite being within the same cortical layer. Interestingly in *Ctip2^–/–^* mice CSMN axons show defects in fasciculation, outgrowth, pathfinding, and abnormal developmental pruning of corticospinal axons, and fail in the connection to the spinal cord (Arlotta et al., 2005). Then, long-distance growth of the primary axons is mediated by several chemoattractants or repellants (including netrins, semaphorins, the SLIT family, ephrins, and repulsive guidance molecules) that diffuse into the local environment and guide the growing corticospinal axons (Martin, 2005; Harel and Strittmatter, 2006). During their elongation, the axons form numerous collateral branches; the refinement of corticospinal terminations occurs during a protracted postnatal period and includes both pruning of transient terminations and growth to new targets (Martin, 2005). Moreover, during the development, neurons can effectively extend their axons also thanks to innate genetic programs (Seiradake et al., 2016). Among the genes involved, Krüppel-like factor 7 (*KLF7*), signal transducer and activator of transcription 3 (*STAT3*) and Sry-related HMG box 11 (*SOX11*) encode for TFs widely expressed in the embryonic CNS and PNS during periods of axon growth (Puttagunta et al., 2014; Tedeschi and Bradke, 2017).

At adulthood, neurons stop expressing the genes responsible for developmental axon elongation (He and Jin, 2016) and epigenetic changes occur, with many of the TF binding sites that drive axon growth-related genes becoming inaccessible (Fawcett, 2020). Indeed, one major issue in case of spinal cord trauma is the difficulty in triggering axonal regrowth and/or reorganizing damaged or spared descending pathways. In particular, the CST shows very poor regeneration ability, compared to other pathways (as the nigrostriatal, the extrapyramidal, and autonomic pathways) that bear a relatively high capability to regrow (Brecknell et al., 1996; Di Giovanni, 2009).

To further highlight the differences between immature and mature CNS, in 2019 Tsujioka and Yamashita compared the gene expression profiles of neonatal and adult sham or injured spinal cords (pyramidotomy model), by performing RNA-sequencing and qRT-PCR validation on C4-C7 spinal levels. In comparison with adult mice uninjured, in the postnatal spinal cords genes related to axonal growth, cell proliferation, and myelination were upregulated, whereas those related to the immune response were downregulated. After pyramidotomy, some genes responsible for the inflammatory response were upregulated in adult mice, suggesting that these genes might be related to the low sprouting potential in adult mice (Tsujioka and Yamashita, 2019).

Since the developmental processes are well known, the attempt to reactivate them within adult neurons could represent an intriguing approach for enhancing axon regeneration after an injury. For example, adult CST neurons are unable to induce the *Klf7* expression after axon injury, but its overexpression by AAV injection into the murine sensory-motor cortex can trigger both sprouting and axonal regeneration after SCI (Blackmore et al., 2012). Similarly, *Stat3* overexpression, when combined with the activation domain from Herpes simplex virus VP6, significantly improved the neurite outgrowth both *in vitro* on primary cortical neurons and *in vivo* in retinal ganglion cells after optic nerve axon injury (Mehta et al., 2016). Likewise, CST neurons fail to spontaneously upregulate *Sox11* after spinal axon injury, but its forced viral expression at the cortical level induced sprouting and axon regeneration: however, *Sox11* overexpression also caused a reduced dexterity in the injected animals, suggesting that it is important to optimize not only the growth but also the function of regenerated axons (Wang et al., 2015).

The tumor suppressor p53 (encoded by *Tp53*) is also a developmentally regulated TF: when overexpressed by viral vectors in spinal cord hemisected mice, it is able to promote CST sprouting (Floriddia et al., 2012).

On the contrary, mature neurons can express genes that limit axon growth in adulthood, but not in the embryo. This mechanism is necessary to prevent ectopic axon growth and aberrant synapse formation (Hilton and Bradke, 2017; Tedeschi and Bradke, 2017). An example is represented by *Klf4*, a transcriptional repressor of regeneration: when overexpressed *in vitro*, it induces a remarkable neurite outgrowth reduction, whereas its silencing triggers axonal regeneration of retinal ganglion cells after nerve optic nerve injury (Moore et al., 2009).

## Cortical Gene Expression After Spinal Cord Injury

Many studies have investigated the genetic programs triggered in the CSMNs after SCI during the last decades. In 2003, by *in situ* hybridizations, Mason and coll. showed that the expression of a number of growth-associated genes (including *C-Jun/Ap-1, L1cam/Ncaml1, Atf3*, and *Krox2-4/Egr1*) was significantly increased after intracortical axotomy (within the neocortex), but not after an injury to the CST at the spinal level (C3/C4). This suggested that the distance of the injury site from the cell body can influence axotomy-induced gene expression (Mason et al., 2003).

However, more recently, the cortical gene expression changes after thoracic CST transaction were evaluated by microarray analyses using total RNA isolated from rat sensory-motor cortex layers V-VI, 1 to 60 days post-injury (DPI). Despite the distance between the lesion site and the relative sensory-motor cortex area, 521 genes (mainly related to wounding, apoptosis, neurogenesis, and cytoskeletal reorganization) underwent significant regulation, as early as 24 h after injury. The number of modulated genes further increased in the following days, reaching the maximum at 21 DPI. Interestingly, in presence of a local spinal anti-scarring treatment, genes regulating the inhibition of axon growth and impairment of cell survival were attenuated, whereas genes associated with axon outgrowth, cell protection, and neural development were upregulated, compared to untreated animals. Overall, this means that dynamic transcriptional responses are triggered in CSMNs by SCI, and further modulated in response to distant regeneration-promoting treatment (Kruse et al., 2011). On the contrary, other studies have investigated the expression of factors limiting axonal regeneration. By performing an *in vitro* genome-wide loss-of-function screening on isolated injured cortical neurons, Sekine and coll. identified many genes involved in transport, receptor binding, and cytokine signaling pathways. Interestingly, *Rab27b* was highly enriched and its lack in injured mice (optic nerve crush) assured a remarkable axonal regeneration (Sekine et al., 2018).

Despite the mentioned limited CNS capability in axonal regrowth after injury, modest levels of spontaneous functional recovery can be observed after trauma, probably due to the plasticity of intact circuitry. By performing a comparison between the transcriptomic profiles of adult murine intact “sprouting” CSMNs in active growth mode with intact “quiescent” CSMNs after pyramidotomy, Fink et al. (2017) identified some pro-axonal growth pathways able to drive functional plasticity within intact spinal circuits after partial SCI: in particular, lipid phosphate phosphatase-related protein type 1 (PLPPR1) and LPAR1 act as intrinsic axonal growth modulators for intact CSMNs after adjacent injury (Fink et al., 2017).

Unlike the CNS, PNS maintains a high regenerative ability during the entire individual lifespan, since after peripheral neuron axotomy hundreds of regeneration-associated genes (RAGs) can be activated (van Kesteren et al., 2011). A typical RAG response involves several hundred genes, including TFs (as *Stat3*, *Sox11*, *c-Jun*, already mentioned) or effector RAGs (as *Gap43, Cap23, Scg10, Npy*), that can successfully support axonal regrowth (Tedeschi, 2012; Ma and Willis, 2015). Instead, in central neurons, a very limited or no RAG-response is observed. However, by acting on the signaling pathways active in the PNS, it is possible to induce GAP43 expression and sustain axonal regrowth also at the central level. For example, IL-6 treatment after SCI was shown to activate the *Jak/Stat3* and *PI3K/Akt* pathways, and in turn upregulate GAP43, promoting neurite outgrowth *in vitro* and synaptogenesis *in vivo*. Similarly, the administration of TDZD-8 (a GSK-3 inhibitor) after SCI is able to increase the GAP43 expression, increase the density of cortical spinal tract fibers at the injury site, and improve the motor performance of SCI rats (Lei et al., 2019). On the other hand, by inhibiting the RhoA kinase activation, the administration of the natural compound β-Elemene can enhance GAP43 expression and neurite outgrowth in SCI rats (Wang et al., 2018). Overall, this means that many molecular cascades converge on GAP43, which clearly represents a crucial target for axonal regeneration in the CNS too.

## MiRNAs

MiRNAs are small non-coding RNAs, which negatively regulate gene expression at post-transcriptional level. In case of SCI, they can cooperate in influencing the molecular pathways regulating axon regeneration as well as inflammation, apoptosis, and remyelination (Ghibaudi et al., 2017). The activation/regulation of miRNAs can also occur at the level of the sensory-motor cortex where the cell bodies of CSMNs are located.

During the last years, both *in vitro* and *in vivo* studies have been performed to unravel the role of the miRNA network in this scenario. For example, miR-20a and miR-128 were able to induce the axon outgrowth of the cultured cortical neurons by regulating the PSD-95/Dlg/ZO-1 homology-Rho guanine nucleotide exchange factor (PDZ-RhoGEF)/Ras homolog gene family member A (RhoA)/GAP43 axis (Sun et al., 2013). Moreover, miR-20a plays a role in SCI-induced neuronal apoptosis through repression on the anti-apoptotic Mcl-1, as demonstrated both *in vitro* (in Neuro-2A neuroblastoma cell line) and *in vivo* (contusive SCI model) (Liu et al., 2015). It can also regulate *Stat3* (see above), although until now this function has been only demonstrated during early embryonic branching morphogenesis in the lung (Carraro et al., 2009). Interestingly, these studies highlight the multiple functional roles of miR-20, also in case of SCI.

The *Stat3/Gap43* pathway is targeted also by miR-17-5p: indeed the *in vitro* downregulation of miR-17-5p is able to promote the axon regeneration of the cortical neurons, suggesting that this miRNA may represent another interesting target for SCI (Zhang et al., 2020).

At the cortical level, miRNAs can also regulate other functions in SCI, apparently not directly correlated with axonal regrowth, such as neuroprotection. For example, after a spinal cord transection at C6 level in mice, miR-7b-3p is significantly upregulated in the sensory-motor cortex. Moreover, *in vitro* and *in vivo* experiments demonstrated that this miRNA can exert a dual role, in the attempt to maintain the axotomized CSMNs more plastic on one side, and to protect them from apoptotic death on the other. Indeed the hypothesis is that increasing the expression of miR-7b-3p after SCI could stimulate the reactivation of developmental programs silenced in adult upper MNs, meanwhile supporting their survival (Ghibaudi et al., 2021).

## Regeneration in Invertebrates and Lower Vertebrates

Unlike mammals, axonal regeneration spontaneously occurs in some invertebrates (as the nematode *Caenorhabditis elegans*, the crustacean *Parhyale hawaiensis*, and the cephalopod *Octopus vulgaris*) and lower vertebrates (including the axolotl *Ambystoma mexicanum* and the lamprey *Petromyzon marinus*), assuring a substantial recovery of locomotor function after SCI. This successful response is due to the activation of mechanisms for axonal elongation and selection of appropriate postsynaptic targets, together with limited necrosis at the injury site, and a permissive environment at the spinal cord level (McClellan, 1998; Agata and Inoue, 2012). The absence of glial scar and astrocyte activation can be relevant to support the regeneration of descending axons, as demonstrated in salamanders (Ryczko et al., 2020). Interestingly, the descending pathways mainly originate from cephalic ganglia, diencephalon, mesencephalon, or rhombencephalon (depending on the species): this can further justify why, unlike the tracts originating from the cerebral cortex, the subcortical descending tracts evolutionarily bear a relatively higher capability to regrow in humans.

Moreover, although a combination of factors seems necessary to facilitate fiber regeneration in some lower vertebrates and invertebrates, it is evident that the neuronal genetic programs are fundamental in these species as well. However, it remains unclear why turning off the developmental processes responsible for regeneration and plasticity has represented an evolutionary step forward for the higher vertebrates. Some theories justify this apparent contradiction with the high energy demanding process for a complex hard-wired nervous system (Fawcett, 2020). Moreover, studying the regenerative capabilities of invertebrates, in the attempt to identify orthologs RAGs, could represent an intriguing approach for stimulating silenced evolutionary neural regeneration pathways in adult mammalian injured CNS.

## Conclusion

In the last years, remarkable progress has been made to understand the mechanisms involved in the CSMN axonal degeneration and “tentative” regeneration after SCI. However, deciphering the genetic differences between development and adult (re)generation remains elusive: some well-known genes (as *KLF7*, *SOX11*, *STAT3*) are active during development and represent potential therapeutic targets in adulthood, whereas in the adult CNS additional genes/TFs/miRNAs can be involved (Figure 1).

It should be also mentioned that, although recapitulating some developmental aspects and involving similar genes/pathways (as *STAT3*), these two processes differ, in part conditioned by other extrinsic aspects. In case of SCI, the environment is hostile and several inhibitory mechanisms can contribute to limit the intrinsic regenerative attempt of CSMN axons. Among the most known inhibitors deeply studied in the last years, we can mention NogoA, chondroitin sulfate proteoglycans, myelin-associated glycoprotein, oligodendrocyte myelin glycoprotein, semaphorin 3A, and tenascin-C (Fawcett, 2020). Therefore, the activation of TFs and miRNAs (as enhancers to drive the regeneration program) could be not enough after a CNS injury. To induce and sustain a substantial axonal regrowth, combined therapeutic approaches are probably needed, both by limiting the potential inhibitory mechanisms and activating transcriptional programs in the axotomized neurons.

With modern experimental approaches, discriminating the different “players” involved in regeneration should be easier. Although many challenges remain, the current technological advances will allow providing new insights into how axonal regrowth is promoted, possibly even exploiting the silenced developmental processes. For example, the combination of multi-layer omics (epigenomics, transcriptomics, proteomics…) and computational methods will help to study axon regeneration mechanisms and rebuild injured neural circuitries (Tedeschi and Popovich, 2019). Moreover, genetic reprogramming (to rejuvenate mature neurons) can represent another interesting strategy [e.g., the forced viral expression of *SOX11* promoted CST sprouting and regrowth in both acute and chronic injury models (Wang et al., 2015)]. Other modern approaches (including circuit-specific genetic technologies, DREADDs, bioengineered rabies) can assess the succeeded axonal regrowth and functional connectivity after SCI (Hilton and Bradke, 2017).

Moreover, in addition to corticospinal projections, other descending pathways should be considered for regeneration, such as extrapyramidal and autonomic pathways. For instance, the raphespinal and the rubrospinal seem to be more plastic in the adult than the CST. In fact, growth-related genes (*c-JUN, Galectin-1, beta-II-Tubulin*) are upregulated in raphe and red nuclei, but not in upper motor neurons (Di Giovanni, 2009). Their regrowth, even though not sufficient to elicit voluntary movements, could support automatic circuits in the spinal cord and improve movements. Moreover, also ascending pathways should be recovered to provide a sensory feedback to supraspinal and spinal motor circuits.

As an additional consideration, we believe that, to truly overcome CNS injury, we still need to increase our knowledge. Indeed, until now, in the SCI field, the researchers have mainly investigated the expression of genes and non-coding RNAs at the injury site, often disregarding the cerebral cortex where CSMNs reside. Of course, these studies have been pivotal to unravel pathogenetic events occurring after an injury (related to local cell death, inflammation, oxidative stress, demyelination, and the inhibitory mechanisms): nevertheless, it is mandatory to further investigate the transcriptional and structural remodeling occurring within the sensory-motor cortex, without neglecting the significant impact of CSMN axotomy on the whole regenerative process.

In conclusion, the review aimed to highlight the complexity of the genetic system orchestrating the central axon (re)generation: we are probably looking only at the tip of the iceberg, just starting now to identify some of the main key players involved. Interestingly the *Gap43* expression can be modulated by the TF Stat3, which in turn can be targeted by some miRNAs (e.g., miR-20a and miR-17-5p): this is an interesting converging pathway, currently representing one of the most promising potential therapeutic targets in the SCI field, since different molecules able to modulate its activity are already available.

## Author Contributions

MB and AV conceived the manuscript. MB wrote the review. AV revised the manuscript. Both authors contributed to the article and approved the submitted version.

## Conflict of Interest

The authors declare that the research was conducted in the absence of any commercial or financial relationships that could be construed as a potential conflict of interest.

## Publisher’s Note

All claims expressed in this article are solely those of the authors and do not necessarily represent those of their affiliated organizations, or those of the publisher, the editors and the reviewers. Any product that may be evaluated in this article, or claim that may be made by its manufacturer, is not guaranteed or endorsed by the publisher.
